# Malignant pleural mesothelioma co-opts BCL-X_L_ and autophagy to escape apoptosis

**DOI:** 10.1038/s41419-021-03668-x

**Published:** 2021-04-15

**Authors:** Duo Xu, Shun-Qing Liang, Zhang Yang, Haitang Yang, Rémy Bruggmann, Simone Oberhaensli, Sabina Berezowska, Thomas M. Marti, Sean R. R. Hall, Patrick Dorn, Gregor J. Kocher, Ralph A. Schmid, Ren-Wang Peng

**Affiliations:** 1 Division of General Thoracic Surgery, Inselspital, Bern University Hospital, Bern, Switzerland; 2 Department for BioMedical Research (DBMR), University of Bern, Bern, Switzerland; 3 Interfaculty Bioinformatics Unit and Swiss Institute of Bioinformatics, University of Bern, Bern, Switzerland; 4 Institute of Pathology, University of Bern, Bern, Switzerland

**Keywords:** Apoptosis, Target identification, Mesothelioma

## Abstract

Escape from programmed cell death is a hallmark of cancer. In this study, we investigated the anti-apoptotic mechanisms and explored the therapeutic potential of BCL-2 homology domain-3 (BH3) mimetics in malignant pleural mesothelioma (MPM), a lethal thoracic malignancy with an extreme dearth of treatment options. By implementing integrated analysis of functional genomic data of MPM cells and quantitative proteomics of patients’ tumors, we identified BCL-X_L_ as an anti-apoptotic driver that is overexpressed and confers an oncogenic dependency in MPM. MPM cells harboring genetic alterations that inactivate the NF2/LATS1/2 signaling are associated with increased sensitivity to A-1155463, a BCL-X_L_-selective BH3 mimetic. Importantly, BCL-X_L_ inhibition elicits protective autophagy, and concomitant blockade of BCL-X_L_ and autophagic machinery with A-1155463 and hydroxychloroquine (HCQ), the US Food and Drug Administration (FDA)-approved autophagy inhibitor, synergistically enhances anti-MPM effects in vitro and in vivo. Together, our work delineates the molecular basis underlying resistance to apoptosis and uncovers an evasive mechanism that limits response to BH3 mimetics in MPM, suggesting a novel strategy to target this aggressive disease.

## Introduction

Malignant pleural mesothelioma (MPM) is a highly aggressive malignancy that is etiologically associated with asbestos exposure^1,2^. Despite the restriction of asbestos use in most countries, the incidence of MPM is still rising due in part to the long latency (around 40 years) of the interval from carcinogen exposure to tumor onset^3^. There are no typical clinical symptoms of mesothelioma in the early phase, and the majority of patients (80%) are diagnosed at advanced stages associated with extremely poor prognosis^4^. Previous studies in MPM have revealed frequent oncogenic events enabled by genetic alterations that inactivate tumor suppressor genes, most often BRCA1 associated protein-1 (*BAP1*), neurofibromatosis type 2 (*NF2*), large tumor suppressor kinase 2 (*LATS2*), and cyclin-dependent kinase inhibitor 2 A/2B (*CDKN2A/2B*), which, however, have proven difficult to be therapeutically exploited^2,5,6^. Further exacerbating the dilemma, platinum-based chemotherapy, the current standard of care for inoperable late-stage MPM, only marginally improves patient survival^7^. Hence, there is a pressing need to identify new druggable targets in MPM and develop effective therapeutic strategies for the daunting disease.

The *NF2* tumor suppressor gene encodes Merlin (Moesin-ezrin-radixin-like protein), which mediates tumor suppression and contact-dependent inhibition by repressing Hippo, mTORC1, RAS, EGFR, and FAK-Src signaling pathways^8^. The Hippo signaling, an evolutionally conserved pathway that regulates organ size and tissue homeostasis by restricting cell growth and promoting apoptosis, is one of the best characterized Merlin/NF2-regulated pathways^9^. Besides the mutation in *NF2*, other components of the Hippo pathway, e.g., large tumor suppressor kinase 1/2 (*LATS1/2*), are also frequently inactivated in MPM patients^6^. Dysregulation of the Hippo pathway constitutively activates Yes-associated protein (YAP), a transcription regulator that promotes the transcription of genes involved in cell proliferation and anti-apoptosis by interaction with TEA/ATTS domain (TEAD) transcription factors^10^.

Resistance to apoptosis, a critical barrier of tumor development, is one of the most prominent hallmarks of cancer^11^. Overexpression of pro-survival B-cell lymphoma 2 (BCL-2) family members (BCL-2, BCL-X_L_, MCL-1, BCL-W,BCL-B, and BFL-1) is a key apoptosis evasion mechanism that promotes tumor growth by keeping pro-apoptosis effectors (BAX/BAK) in check^11^. By contrast, the BCL-2 homology domain-3 (BH3)-only proteins (BAD, BIM, BID, NOXA, PUMA, BIK, BMF, and HRK) induce apoptosis by neutralizing the pro-survival BCL-2 proteins^12^. As such, targeting anti-apoptotic regulators with BH3 mimetics represents an attractive strategy for cancer therapy^13^. Several BH3 mimetics, e.g., the BCL-2/BCL-X_L_/BCL-W inhibitor ABT-263 (navitoclax), BCL-2–selective inhibitor venetoclax (ABT-199), and BCL-X_L_–selective inhibitor A-1155463, have showed promising clinical activity^14^. In particular, venetoclax has been approved by the US Food and Drug Administration (FDA) for the treatment of chronic lymphocytic leukemia (CLL) with a 17p-deletion or TP53 mutation^15^. We and others have reported that MPM cells can acquire anti-apoptotic adaptation as a protective mechanism to evade oncogenic stress and anticancer therapy^16–18^. In this study, we systematically analyzed the cell survival dependency on anti-apoptotic BCL-2 proteins and explored the potential of specific BH3 mimetics as anti-MPM therapy.

## Materials and methods

### Cell culture and reagents

Human normal mesothelial cells (LP-9) was a gift from Robert Kratzke (Masonic Cancer Center, University of Minnesota, USA)^19^ and cultured in Medium 199 (Cat. #M7528; Sigma-Aldrich) supplemented with 15% fetal bovine serum (Cat. #10270-106; Life Technologies), 1% penicillin/streptomycin solution (Cat. #P0781, Sigma-Aldrich), 10 ng/ml of epidermal growth factor (Cat. #E5036, Sigma-Aldrich), and 0.4 μg/ml hydrocortisone (Cat. #07904, STEMCELL TECHNOLOGIES) at 37 °C with 95% air/5% CO_2._ Human normal lung fibroblasts (hFb16Lu;CCD-16Lu) and human MPM cell lines (MESO-1, MESO-4, JL-1, H2452, MSTO-211H, and H2052) were previously described^18,20^. All cells were authenticated by short tandem repeat (STR) profiling and regularly tested free of mycoplasma (Microsynth). A-1155463 (Cat. #CS-5398), hydroxychloroquine (Cat. #CS-8017), Venetoclax (Cat. #S8048), and ABT-263 (Cat. #S1001) were obtained from ChemScene and SelleckChem, respectively. Bafilomycin A1 was provided by K. Krempaska (Department for Biomedical Research, University of Bern, Switzerland).

### Cell viability and clonogenic survival assay

Cell viability was measured by acid phosphatase (APH) assay as described^18,20^. Each data point was generated in triplicate from three independent experiments (*n* = 3). IC_50_ values were determined based on the best-fit curve generated in GraphPad Prism [log (inhibitor) vs. normalized response]. Combination Index (CI) was calculated by ComboSyn software^21^. CI < 1.0, synergism; CI = 1.0, additive effects, CI > 1.0, antagonism. Clonogenic assay was done as described^18,20^. Briefly, cells seeded in 6-well plates (1000–2000 cells/well) were treated for 96 h and cultured in the absence of drugs for 10–12 days depending on growth rate. The resulting colonies were stained with crystal violet (0.5% dissolved in 25% methanol).

### Immunoblotting and immunohistochemistry

Cell lysates were prepared and immunoblot analysis was performed as described^18,22^. In brief, protein lysates were resolved by SDS-PAGE (Cat. #4561033; Bio-Rad Laboratories) and transferred onto nitrocellulose membranes (Cat. #170-4158; Bio-Rad). After incubation with blocking buffer (Cat. #927-4000; Li-COR Biosciences) for 1 h at room temperature, membranes were incubated with primary antibodies (BCL-X_L_: 1:1000, #2764; Cleaved Caspase-7: 1:1000, #9491; LC3B: 1:500, #12741; Beclin-1: 1:1000, #3495; p62: 1:500, #5114; ATG5:1:1000, #12994; Cell Signaling Technology) overnight at 4 °C. IRDye 680LT-conjugated goat anti-mouse IgG (Cat. #926-68020) and IRDye 800CW-conjugated goat anti-rabbit IgG (Cat. #926-32211) from Li-COR Biosciences were used at 1:10000 dilutions. Signals of membrane-bound secondary antibodies were visualized by the Odyssey Infrared Imaging System (Li-COR Biosciences), followed by quantification using Image J^23^.

Immunohistochemical study were performed as described^24^. In brief, surgically removed xenograft tumors (two tumors/group) were formalin-fixed, paraffin-embedded (FFPE), and stained with hematoxylin and eosin (H&E) using standard protocols. FFPE tissue blocks were sectioned at 4 μm, deparaffinized, rehydrated, and subsequently stained with appropriate antibodies (LC3B: 1:4000, #3868, Cell Signaling Technology; p62: 1:8000, # WH0008878M1, Sigma; Cleaved Caspase-3: 1:200; # 9664, Cell Signaling Technology) using the automated system BOND RX (Leica Biosystems)^24^. Visualization was performed using the Bond Polymer Refine Detection kit (Leica Biosystems) as instructed by the manufacturer. Images were acquired using PANNORAMIC^®^ whole slide scanners and processed using Case Viewer (3DHISTECH Ltd.). The staining intensities of the whole slide (two tumors/group) were quantified by QuPath software^25^.

### Apoptosis assay

MPM cells were treated as specified in the figure legends. After treatment, cells in the supernatant and adherent to plates were collected, washed with PBS, and pooled before suspended in 400 μl binding buffer and stained with the Annexin V Apoptosis Detection Kit-FITC (Cat. #88-8005; Thermo Fisher Scientific) according to the manufacturer’s instructions. Flow cytometry analysis was performed on a BD Biosciences LSRII flow cytometer.

### Autophagic flux assay

The mCherry-eGFP-LC3B lentivirus was kindly provided by Mario P. Tschan (Institute of Pathology, University of Bern, Switzerland). Briefly, lentivirus was transduced into cell lines followed by selection with puromycin (1 μg/ml) for 3 days and various treatments. Cells were then trypsinized and resuspended for flow cytometry analysis of GFP and mCherry fluorescence using a BD Biosciences LSRII flow cytometer. Data were analyzed by FlowJo software and gates for populations with low/intermediate/high mCherry/GFP ratio were set according to previous studies^26,27^.

### Small interfering RNA (siRNA) knockdown

Knockdown of *BCL2L1* and *ATG5* was achieved by specific duplex siRNAs (*BCL2L1* siRNA, 15 nM; *ATG5* siRNA, 30 nM) purchased from Origene Technologies (Cat. #SR319459 and SR322789). Transfection of siRNAs was performed with Lipofectamine 2000 (Cat. #116628027, Invitrogen) according to the manufacturer’s instructions.

### Animal experiments

Mouse experiments were conducted in accordance with Institutional Animal Care and Ethical Committee-approved animal guidelines and protocols. Experiments were performed in 8-week-old male NSG (NOD-*scid IL2Rγ*^*null*^) mice, with sample size not predetermined by statistical method but rather based on preliminary experiments. Group allocation was performed in a randomized but not blinded manner. Suspensions of MESO-1 cells mixed 1:1 with Matrigel (Cat. #356231; Corning) were subcutaneously inoculated in the flanks (1 × 10^6^ cells /injection). One month after injection, mice were randomly assigned to treatment groups (*n* = 5): (1) control; (2) A-1155463 (5 mg/kg, i.p., once daily); (3) HCQ (50 mg/kg, i.p., once daily); (4) combination of A-1155463 and HCQ in the abovementioned doses(Tumor size was measured by digital caliper every two to three days. Tumor volume was calculated as follows: (length x width x width)/2. Mice were sacrificed at the end of 23-day treatment.

### Public databases (TCGA, TCPA, CBioPortal, DepMap, GEO, GSDC, and COSMIC)

Interrogation of publicly available dataset was performed as we have described^28^. Specifically, transcriptome profiling and reverse-phase protein array data of mesothelioma patients were obtained from the Cancer Genome Atlas (TCGA), the Cancer Proteome Atlas (TCPA), and the cBio Cancer Genomics Portal (CBioPortal)^29,30^. The catalog of gene essentiality across MPM cell lines is obtained from the Cancer Dependency Map Project (DepMap)^31^. Transcriptomic data of MPM samples (GSE2549) was downloaded from the Gene Expression Omnibus(GEO)^32^. Genomics of Drug Sensitivity in Cancer (GDSC) and Catalog of Somatic Mutations in Cancer (COSMIC) was used to extract transcriptomic data and mutation status of MPM cell lines^33,34^.

### Statistical analysis

Statistical analyses were performed using GraphPad Prism 8 (GraphPad Software, Inc.). All samples that met proper experimental conditions were included in the analysis. Data represent biological replicates (*n*) and are depicted as mean values ± s.d. or mean values ± SEM as indicated in the figure legends. Comparison of mean values was conducted with unpaired, two-tailed Student’s *t*-test, one-way or two-way ANOVA as indicated in the figure legends, *P* < 0.05 were considered statistically significant.

## Results

### BCL-X_L_ is deregulated and confers a survival dependency in MPM

**Fig. 1:**
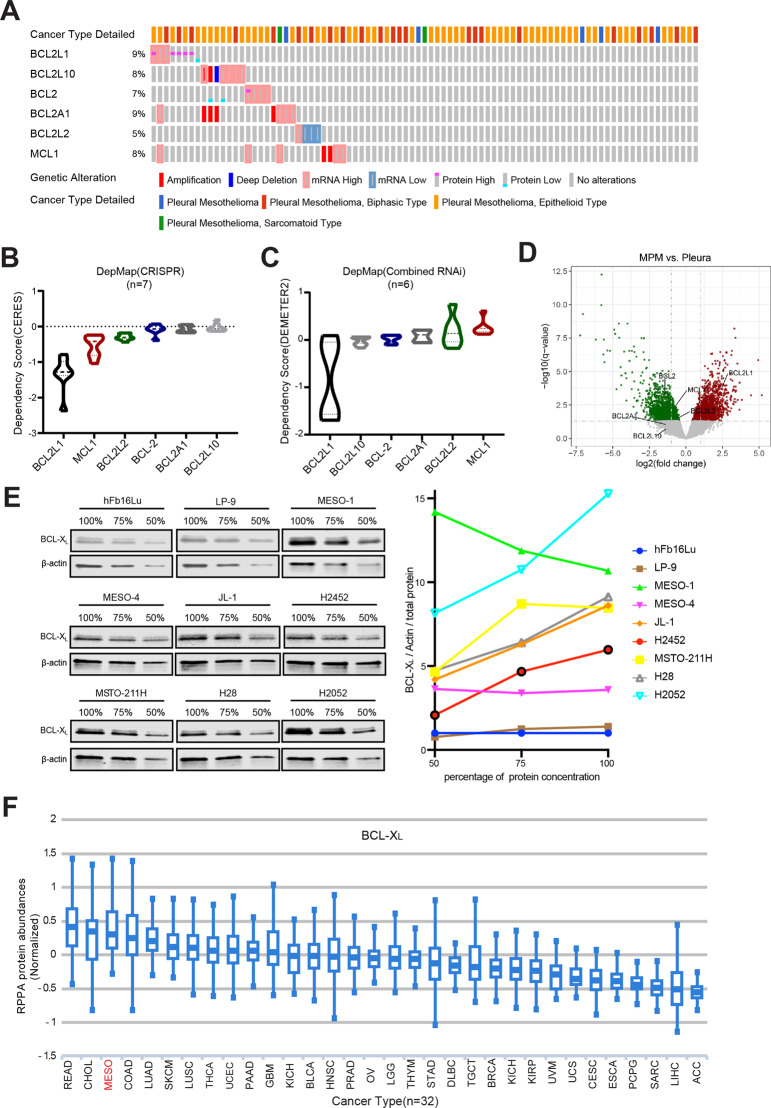
BCL-X_L_ is overexpressed and confers a survival dependency in MPM. **A** Genetic alterations and mRNA/protein expression of pro-survival Bcl-2 family genes in the TCGA cohort of MPM patients (*n* = 87). Data were downloaded from the cBio Cancer Genomics Portal (CBioPortal). **B**, **C** The dependency profile of pro-survival BCL-2 family genes (*BCL2L1, BCL2L10, BCL2, BCL2A1, BCL2L2,* and *MCL1*) in MPM cell lines based on genome-wide CRISPR (NCIH28, NCIH2452, MPP89, NCIH2052, ISTMES2, MERO14, and MSTO-211H) or RNAi interference (MPP89, ACCMESO-1, NCI-H28, NCIH2452, NCI-H2052, and JL-1) screens. Data were downloaded from The Cancer Dependency Map Project (DepMap) datasets: CRISPR (Avana) Public 19Q3 and Combined RNAi (Broad, Novartis, Marcotte). **D** Volcano plot of transcriptomic comparison between patients’ MPM samples (*n* = 40) versus normal pleural tissues (*n* = 5) (GSE2549). Genes significantly downregulated (adjusted *p* value <0.05) are shown in green and genes significantly upregulated in red. The differentially expressed pro-survival Bcl-2 family genes are highlighted. **E** Immunoblots of BCL-X_L_ in human normal fibroblasts (hFb16Lu), normal mesothelial cells (LP-9), and MPM cells. Proteins lysates prepared from the cells were subjected to serial dilutions (100, 75, and 50%) for immunoblot analysis (left). Densitometric analysis of the immunoblot (right) showed fold change of BCL-X_L_ signal normalized against Actin and the total protein, with the value of hFb16Lu cells set to 1. **F** BCL-X_L_ protein level in TCGA pan-cancer cohort (*n* = 32). The expression profile was obtained from The Cancer Proteome Atlas (TCPA). READ rectum adenocarcinoma, CHOL cholangiocarcinoma, MESO mesothelioma, COAD colon adenocarcinoma, LUAD lung adenocarcinoma, SKCM skin cutaneous melanoma, LUSC lung squamous cell carcinoma, THCA thyroid carcinoma, UCEC uterine corpus endometrial carcinoma, PAAD pancreatic adenocarcinoma, GBM glioblastoma multiforme, KICH kidney chromophobe, BLCA bladder urothelial carcinoma, HNSC head and neck squamous cell carcinoma, PRAD prostate adenocarcinoma, OV ovarian serous cystadenocarcinoma, LGG brain lower grade glioma, THYM thymoma, STAD stomach adenocarcinoma, DLBC lymphoid neoplasm diffuse large B-cell lymphoma, TGCT testicular germ cell tumors, BRCA breast invasive carcinoma, KICH kidney chromophobe, KIRP kidney renal papillary cell carcinoma, UVM uveal melanoma, UCS uterine carcinosarcoma, CESC cervical squamous cell carcinoma and endocervical adenocarcinoma, ESCA esophageal carcinoma, PCPG pheochromocytoma and paraganglioma, SARC sarcoma, LIHC liver hepatocellular carcinoma, ACC adrenocortical carcinoma.

In the attempt to explore the potential of BH3 mimetics as anti-MPM therapy, we investigated genetic status, transcriptional expression, and dependency profile of the pro-survival BCL-2 gene family (*BCL2L1, BCL2L10, BCL2, BCL2A1, BCL2L2,* and *MCL1*) in MPM by interrogating TCGA dataset and functional genomics that determines genetic dependencies in cancers (DepMap; https://depmap.org/portal/). The integrated molecular characterization revealed that, of the anti-apoptotic genes, *BCL2L1* (encoding BCL-X_L_) is altered in a subset of MPM patients (*n* = 87), by means of gene amplification and mRNA overexpression (Fig. 1A) and MPM cells show the greatest dependency on *BCL2L1* for survival (Fig. 1B, C). Consistently, *BCL2L1* expression is significantly upregulated in patients’ MPM compared with that in normal pleural tissues (Fig. 1D). Our immunoblot analysis revealed upregulated expression of BCL-X_L_ in human MPM cell lines compared with normal lung fibroblasts (hFb16Lu) and mesothelial LP-9 cells (Supplementary Fig. 1). Notably, a remarkably greater increase in BCL-X_L_ was observed in MPM cells compared with normal controls when BCL-X_L_ signal was normalized against Actin (loading control) and the total protein (Fig. 1E). Further supporting these observations, examination of TCPA dataset, which provides quantitative proteomics of patient-derived pan-cancers (*n* = 32), revealed that MPM had the third highest level of BCL-X_L_ (Fig. 1F). These results indicate that anti-apoptotic BCL-X_L_ is deregulated in MPM at genetic, transcriptional, and translational levels.

**Fig. 2:**
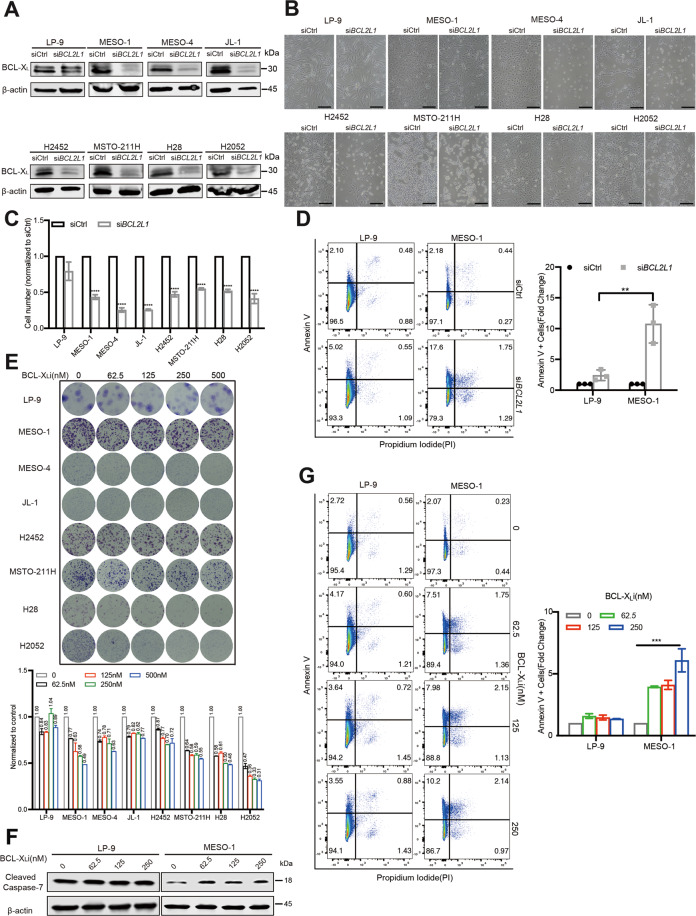
Genetic and pharmacological inhibition of BCL-X_L_ preferentially impairs MPM cell proliferation. **A-C** Immunoblots (**A**) and micrographic images (**B**) of LP-9 and MPM cells with siRNA-based *BCL2L1* knockdown. Viable cells were counted 48 h post-transfection by trypan blue dye exclusion (**C**). Data were presented as mean ± s.d. (*n* = 3). ****p < 0.0001 by two-way ANOVA with Tukey’s multiple comparisons test. Scale bar: 50 μm. **D** Flow cytometry-based apoptotic assay of LP-9 and MESO-1 cells 48 h post-transfection with siRNAs. Shown on the right is fold change in apoptotic cells (Annexin V-positive) induced by *BCL2L1* knockdown (si*BCL2L1*) compared with control siRNA (siCtrl), with the value of the control treatment set as 1. Data were presented as mean ± s.d. (*n* = 3), with a representative plot shown in the left. **p < 0.01 by two-way ANOVA with Tukey’s multiple comparisons test. **E** Clonogenic assay of LP-9 and MPM cells treated with A-1155463 for 96 h and subsequently cultured in the absence of the drug for additional 12 days. Quantification is shown underneath, with data presented as mean ± s.d. (*n* = 2). **F** Immunoblots of LP-9 and MESO-1 cells treated with A-1155463 for 24 h. **G** Flow cytometry-based apoptotic assay of LP-9 and MESO-1 cells after treatment with A-1155463 for 48 h. Shown on the right is fold change in apoptotic cells (Annexin V-positive) induced by treatment with A-1155463 treatment (BCL-X_L_i) compared with vehicle control (0 nM), with the value of the control treatment set as 1. Data were presented as mean ± s.d. (*n* = 3), with a representative plot shown in the left. ***p < 0.001 by two-way ANOVA with Tukey’s multiple comparisons test.

Next, we addressed whether *BCL2L1* represents a genetic vulnerability in MPM. *BCL2L1* knockdown by siRNAs caused significantly greater proliferative inhibition and apoptotic cell death in MPM cells (MESO-1, MESO-4, JL-1, H2452, MSTO-211H, H28, and H2052) than in LP-9 cells (Fig. 2A–D). Consistent with the genetic results, A-1155463, a potent and highly selective BCL-X_L_ inhibitor^14^, preferentially impaired MPM cells, resulting in significantly greater growth inhibition in MPM cells than in LP-9 cells (Fig. 2E). Importantly, A-1155463 induced cleavage of caspase-7 (Fig. 2F) and a dose-dependent increase of apoptotic cells in MESO-1, as manifested by flow cytometry-based apoptotic analysis, which showed that treatment with 62.5, 125, and 250 nm A-1155463 resulted in 4-, 4.4-, and 5.4-fold increases in apoptotic cells (Annexin V-positive) compared with vehicle treatment (Fig. 2G). In contrast, A-1155463 treatment barely increased apoptosis compared with vehicle control in LP-9 cells (Fig. 2F, G). Importantly, the selective BCL-2 inhibitor Venetoclax failed to distinguish malignant from normal mesothelial cells, leading to almost equal effects on MPM and LP-9 cells (Supplementary Fig. 2A). Taken together, these results reveal that BCL-X_L_ is highly deregulated and confers an oncogenic dependency in MPM.

### *NF2/LAST1/2* mutations are associated with increased sensitivity to BCL-X_L_ inhibition in MPM

**Fig. 3:**
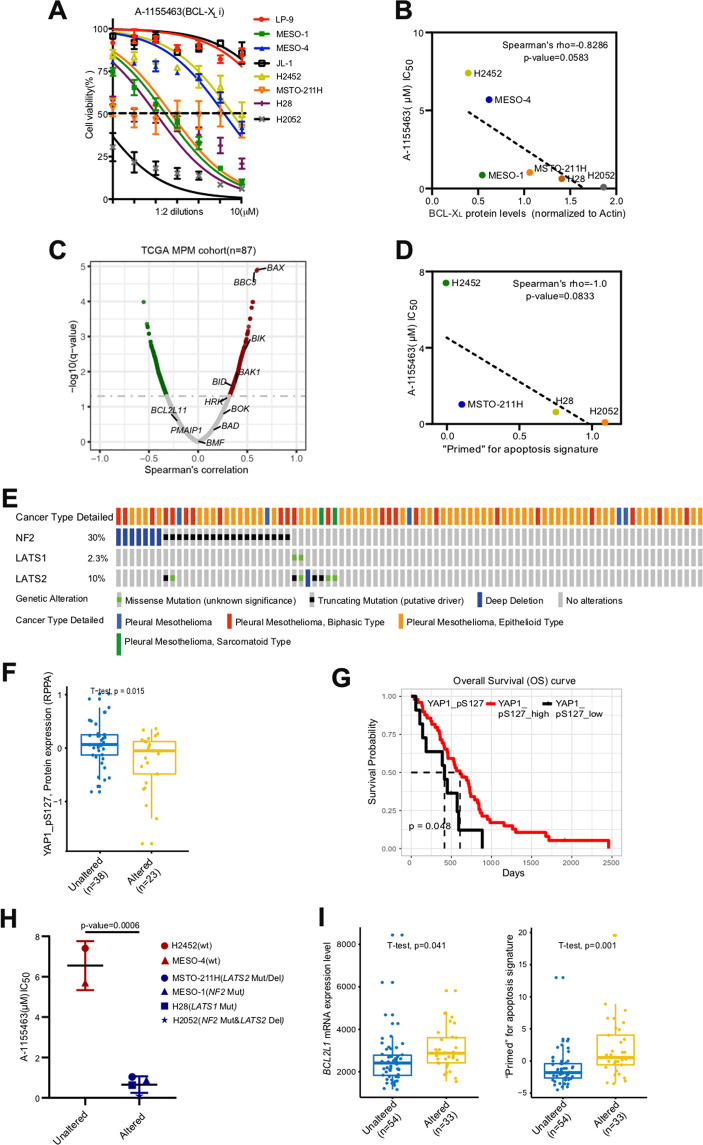
*NF2/LAST1/2* mutations are associated with increased sensitivity to BCL-X_L_ inhibition in MPM cells. **A** Cell viability assay of LP-9 and a panel of MPM cell lines treated with A-1155463 for 96 h. Data were presented as mean ± s.d. (*n* = 3). **B** Correlation analysis of BCL-X_L_ protein levels and the 50% inhibitory concentration (IC_50_) values of A-1155463 in MPM cell lines. **C** Volcano plot of Spearmans’ rank correlation coefficient between gene expression of *BCL2L1* and that of pro-apoptotic genes in patients’ MPM samples (*n* = 87). Data were downloaded from TCGA and subsequently analyzed by R software (Cor.test function). **D** Correlation analysis of “primed” for apoptosis signature (based on the sum of gene expression of *BAK1*, *BAX*, *BBC3*, *BID*, *BIK*, *BOK*, and *HRK*) and the IC_50_ of A-1155463 in MPM cell lines. Transcriptomic data of the indicated MPM cell lines were extracted from Catalog of Somatic Mutations in Cancer (COSMIC). **E** Genetic status of *NF2/LATS1/2* in TCGA cohort of MPM patients (*n* = 87). Data were downloaded from cBioPortal. **F** Genetic alterations in *NF2*/*LATS1*/*2* are associated with decreased protein level of YAP1_pS127. MPM patients (*n* = 61) in a TCGA cohort were stratified according to *NF2*/*LATS1*/*2* genetic status (altered or unaltered), with *p* value calculated by unpaired, two-tailed Student’s *t*-test. **G** Kaplan–Meier curves showing overall survival (OS) of a TCGA cohort of MPM patients (*n* = 61) stratified by protein level of YAP1_pS127 (high versus low). The *p* value is calculated by the log-rank test using R (version 3.4.3). **H** *NF2*/*LATS1*/*2*-mutant MPM cells showed increased sensitivity to A-1155463 (IC_50_)^.^ MPM cells were grouped according to genetic status of *NF2*/*LATS1*/*2*, with *p* value calculated by unpaired, two-tailed Student’s *t*-test. WT, no alterations in *NF2*/*LATS1*/*2*. **I** Genetic alterations in *NF2*/*LATS1*/*2* are associated with increased *BCL2L1* mRNA levels and with a higher gene signature “primed” for apoptosis. MPM patients of a TCGA cohort (*n* = 87) were grouped according to *NF2*/*LATS1*/*2* genetic status in the tumors (altered or unaltered). *p* value was calculated by unpaired, two-tailed Student’s *t*-test.

Next, we sought to identify potential biomarkers associated with MPM response to BCL-X_L_ inhibition in MPM. As expected, the BCL-X_L_ protein level was positively correlated with the sensitivity to A-1155463 [negatively with the IC_50_ (50% inhibitory concentration)] in MPM cells (Fig. 3A, B).

It has been shown that cancer cells express high levels of pro-apoptotic proteins that, however, are constrained via heterodimerization by anti-apoptotic effectors during tumorigeneis^11,35^. As a result, cancer cells can be considered to be ready to undergo apoptosis or “primed for apoptosis”, highlighting the potential of BH3 mimetics in the clinic^36^. To explore a possible link of *BCL2L1* with pro-apoptotic proteins in MPM, we examined a cohort of MPM patients (*n* = 87) in TCGA, which revealed that *BCL2L1* mRNA level was positively correlated with that of several pro-apoptotic genes, e.g., *BAX*, *BBC3*, *BIK*, and *BAK1* (Fig. 3C). Consistent with this observation, MPM cells that overexpress BCL-X_L_ also have higher protein levels of BAX (Supplementary Fig. 2B), suggesting that *BCL2L1-*positive MPM tumors are “primed” to apoptosis induction. By using a previously defined “primed for apoptosis” gene signature, determined by transcriptional expression of the pro-apoptotic genes (*BAK1*, *BAX*, *BBC3*, *BID*, *BIK*, *BOK*, and *HRK*)^37^, we curated the “primed for apoptosis” score of H2452, MSTO-211H, H28, and H2052 cells, chosen for the availability of their transcriptomic data in the Catalog of Somatic Mutations in Cancer (COSMIC). Our analysis revealed that MPM cells with higher scores of the apoptosis gene signature were more sensitive (lower IC_50_ values) to A-1155463 (Fig. 3D), supporting the notion that expression of the pro-apoptotic genes predicts BCL-X_L_ inhibitor sensitivity.

Finally, we explored if recurrent genetic alterations in MPM are associated with sensitivity to BCL-X_L_ inhibition. *NF2/LATS1/2* loss of function (deletion, truncation, and mutation) is frequent (38%) in MPM (Fig. 3E), which downregulates YAP phosphorylation (YAP1_pS127) and increases the activity of YAP oncoprotein (Fig. 3F). Consistent with this observation, MPM patients with low levels of YAP1_pS127 (increased YAP activity) were associated with dismal prognosis (Fig. 3G) and MPM cells harboring *NF2/LATS1/2* mutations/deletions exhibited increased sensitivity to A-1155463 (Fig. 3H). Supporting this finding, examining the TCGA cohort of MPM patients (*n* = 87) showed that *NF2/LATS1/2-*altered tumors were characterized by an increased *BCL2L1* expression and “primed for apoptosis” score (Fig. 3I).

Thus, BCL-X_L_ protein expression, “primed for apoptosis” gene signature and genetic alterations in *NF2/LATS1/2* may serve as biomarkers to stratify MPM subsets that likely benefit from BCL-X_L_ targeted therapy.

### BCL-X_L_ inhibition elicits protective autophagy in MPM cells

**Fig. 4:**
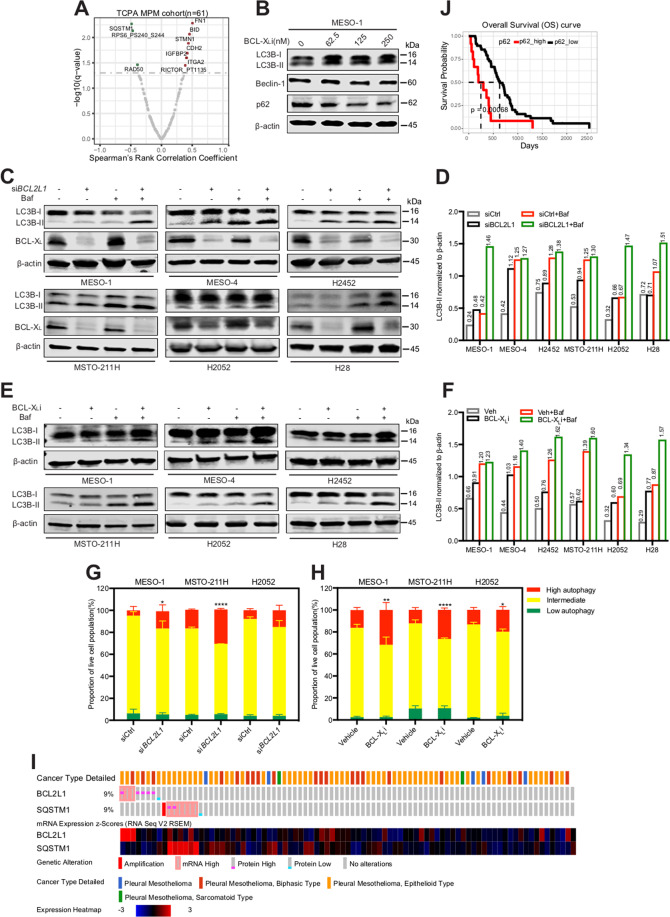
Genetic and pharmacological inhibition of BCL-X_L_ increases autophagic flux in MPM cells. **A**, Volcano plot of Spearmans’ rank correlation coefficient between BCL-X_L_ and other tested proteins (*n* = 217). Proteomic data were downloaded from TCPA, and proteins significantly (adjusted *p* value (or *q* value) <0.05) correlated with BCL-X_L_ are highlighted. **B** Immunoblots of MESO-1 treated with A-1155463 for 24 h. **C**, **D** MPM cells transiently transfected with *BCL2L1*-siRNAs (siBCL2L1) were treated (48 h post-transfection) with Bafilomycin (200 nM) or vehicle (DMSO) for 2 h before subjected to immunoblots to assess autophagic flux. Protein quantification is shown in (**D**). Note the increase in LC3B-II/Actin ratio by *BCL2L1* knockdown in the absence and presence of Bafilomycin. **E**, **F** MPM cells pre-treated with A-1155463 (1 μM) for 24 h were exposed to Bafilomycin (200 nM) or vehicle (DMSO) for another 2 h before immunoblot analysis. Protein quantification is shown in (**F**). Note the increase in LC3B-II/Actin ratio by A-1155463 treatment in the absence and presence of Bafilomycin. **G**, **H** MPM cells stably expressing mCherry-eGFP-LC3B were transfected with *BCL2L1*-siRNA (siBCL2L1) or control siRNA for 48 h (**G**), or treated with A-1155463 (1 μM) or vehicle for 24 h (**H**) before analyzed by flow cytometry. Data were presented as mean ± s.d. (**n** = 3). *p < 0.05, **p < 0.01, and ****p < 0.0001 by two-way ANOVA with Sidak’s multiple comparisons test of control high versus experimental high (red). **I** Genetic status and mRNA/protein expression of *BCL2L1* and *SQSTM1* in a TCGA cohort of MPM patients (*n* = 87). Data were downloaded from cBioPortal. **J** Kaplan–Meier analysis of MPM based on p62 protein level. TCGA cohort of MPM patients (*n* = 61) with high- (in red) or low-p62 (in black) were stratified by optimal cutoff value of the p62 across all patients using the surv_cutpoint function in the R “maxstat” package. The *p* value is calculated by the log-rank test using R (version 3.4.3).

Our observations that MPM cells show heterogeneous responses to BCL-X_L_ inhibition (Figs. 2, 3) suggest the existence of resistance mechanisms. This prompted us to explore the approaches to improve the efficacy of BCL-X_L_-targeted therapy. Interrogation of TCPA dataset revealed SQSTM1 (sequestosome 1; also termed p62) as the top candidate that is significantly negatively correlated with BCL-X_L_ (Fig. 4A). p62 is a key component of autophagic machinery functioning as a cargo adapter by physical interaction with and subsequent delivery of autophagic substrates to autophagosomes for degradation^38^, suggesting a possible role for autophagy to protect MPM cells from the stress elicited by BCL-X_L_ inhibition. Supporting our hypothesis, treatment with A-1155263 acutely increased autophagic activity in MESO-1 cells, marked by decreased p62, upregulated Beclin-1, and microtubule-associated proteins 1A/1B light chain 3B (LC3B)-II (Fig. 4B), whereby the conversion of cytosolic LC3B-I to autophagosome-localized LC3B-II is proportional with initiation of autophagy and therefore serves as a reliable marker of autophagosomes^38^. Moreover, genetic (siRNAs) and pharmacological (A-1155463) inhibition of BCL-X_L_ markedly increased the LC3B-II lapidated form, in particular the ratio of LC3B-II/Actin signal, compared with vehicle controls at the basal level and in the presence of Bafilomycin A1, an inhibitor of autophagosome-lysosome fusion^26,38^, in a panel of MPM cells (Fig. 4C–F). In sharp contrast, the same effects of BCL-X_L_ inhibition were not observed in LP-9 cells, as A-1155463 alone showed no effect on the LC3B-II/Actin ratio (compared with vehicle control), as did concomitant treatment with A-1155463 and Bafilomycin (compared with Bafilomycin alone) (Supplementary Fig. 2C). Importantly, using the mCherry-eGFP-LC3B fluorescence reporter^26,27^, we showed that genetic and pharmacological inhibition of BCL-X_L_ significantly increased the mCherry:GFP fluorescence ratio, a well-recognized measure of autophagic flux, in MPM cells stably expressing mCherry-eGFP-LC3B (Fig. 4G, H), further strengthening the notion that BCL-X_L_ inhibition increases autophagy.

SQSTM1/p62 deregulation (e.g., mRNA upregulation or protein overexpression) occurred in a subset (9%) of MPM patients (Fig. 4I). Although implications of SQSTM1/p62 changes can be context-dependent and deserve cautious interpretations, the steady state level of p62 do reflect the autophagic status^38^ and it is widely accepted that impaired autophagy contributes to initiation and early development of cancer^38,39^. Indeed, previous studies associated decreased p62 or high autophagy status with better clinical outcomes in MPM and other tumors^40,41^. Supporting this notion, high p62 levels predicted poorer prognosis in MPM patients, opposite to the prognostic value of BCL-X_L_ (Fig. 4J, Supplementary Fig. S3A). Moreover, deregulation of SQSTM1/p62 and *BCL2L1* appeared mutually exclusive (Fig. 4I), reiterating the reciprocal nature of p62-and BCL-X_L_-regulated processes in MPM development. Together, these results reveal that BCL-X_L_ inhibition elicits autophagy, which may act as a compensatory mechanism that counteracts BCL-X_L_ targeted therapy.

### Concomitant blockage of BCL-X_L_ and autophagy synergistically enhances anti-MPM effects

**Fig. 5:**
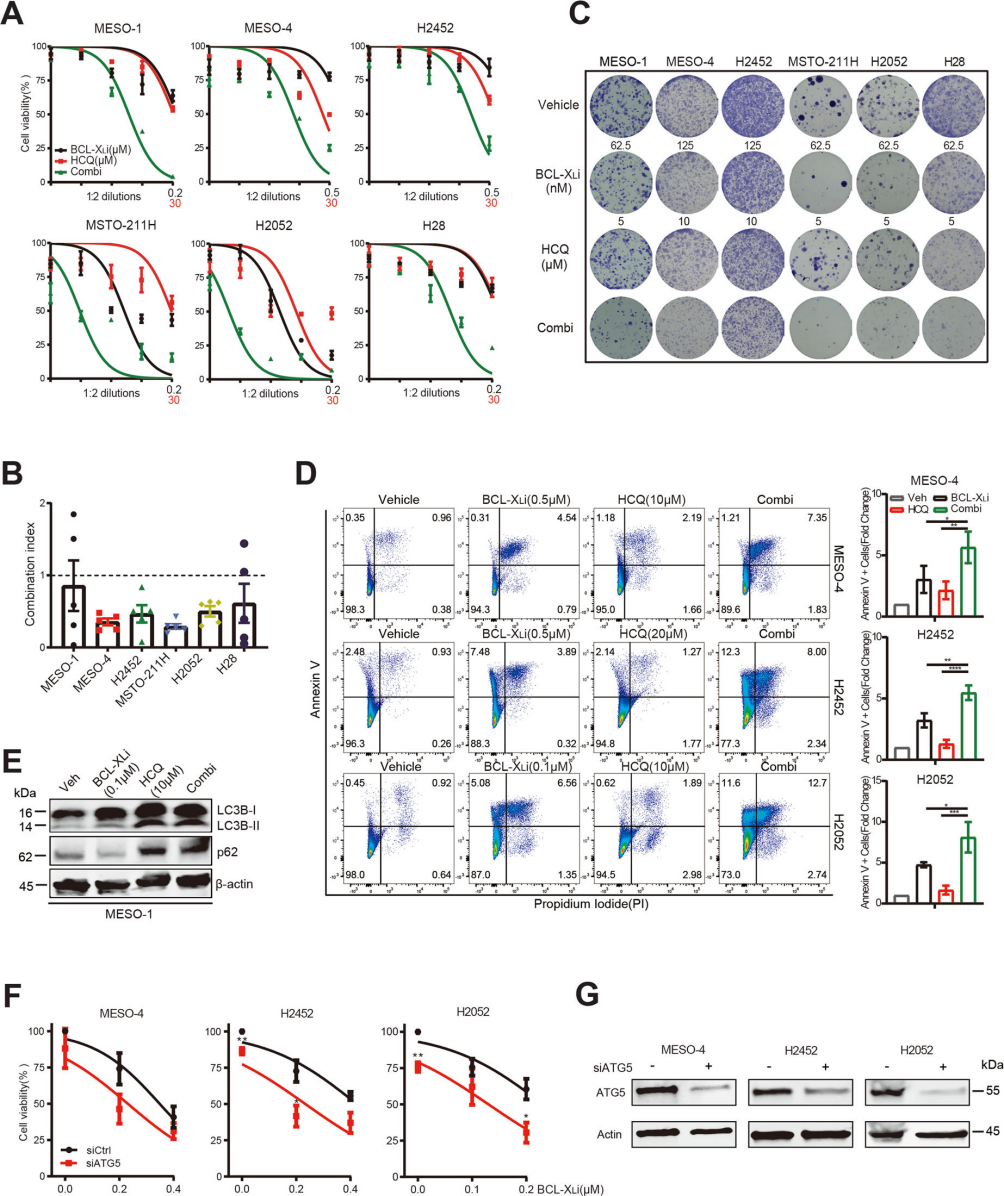
Dual inhibition of BCL-X_L_ and autophagy synergistically enhances antiproliferative effects and increases apoptosis. **A**, **B** Dose-response curves (**A**) of MPM cells treated with A-1155463 and HCQ, alone or in combination, for 96 h. Combination index (CI) values (**B**) were determined by the CompuSyn software. Data were shown as mean ± s.d. (*n* = 3). CI < 1.0, synergism; CI = 1.0, additive effects, CI > 1.0, antagonism. **C** Clonogenic assay of MPM cells treated for 96 h with A-1155463 and HCQ, alone or in combination. Cells were cultured without the drug for additional 12 days before crystal violet staining. Representative images of three independent experiments (*n* = 3) are shown. **D** Flow cytometry-based apoptosis assay of MPM cells treated with A-1155463 and HCQ, alone or in combinations for 48 h. Data were presented as mean ± s.d. (*n* = 3), with a representative plot shown in the left. *p < 0.05, ** *p* < 0.01, ***p < 0.005, and **** *p* < 0.001 by one-way ANOVA with Sidak’s multiple comparisons test. **E** Immunoblots of MESO-1 cells treated with A-1155463 and HCQ, alone and in combination, for 24 h. **F**, **G** MPM cells transfected with *ATG5*- or control siRNAs (siATG5, siCtrl) were analyzed (48 h post-transfection) by immunoblots (**F**) or treated with A-1155463 for another 24 h, followed by quantification of viable cells (**G**). Data were shown as mean ± s.d. (*n* = 3). *p < 0.05 and ** *p* < 0.01 by unpaired *t*-test.

To test this hypothesis that autophagy protects MPM cells from the cytotoxicity of BCL-X_L_ inhibition, MPM cells were concomitantly treated with A-1155463 and the autophagy inhibitor hydroxychloroquine (HCQ) across a broad range of concentrations. While single agents suppressed cell proliferation in a dose-dependent manner, A-1155463 plus HCQ produced a strong synergy, leading to significantly enhanced antiproliferative effects and apoptotic cell death in a panel of MPM cells, including *NF2/LATS1/2*-mutant and wild-type (Fig. 5A–E) but not in human normal lung fibroblasts (Supplementary Fig. S3B). The synergy also applied when HCQ was combined with ABT-263, a pan-inhibitor against BCL-2, BCL-X_L_, and BCL-W (Supplementary Fig. S3C–E).

To further explore the role of autophagy in MPM response to BCL-X_L_ inhibition, we knocked down autophagy related 5 (*ATG5)*, which encodes a key effector protein (ATG5) involved in the initiation of pre-autophagosome formation^39^. Genetic depletion of *ATG5* in MPM cells significantly enhanced the antiproliferative effects of BCL-X_L_ inhibition (Fig. 5F, G), which is in line with the results of pharmacological studies (Fig. 5A–D). Thus, BCL-X_L_ inhibition elicits protective autophagy and combined blockage of BCL-X_L_ and autophagy represents a promising strategy to treat MPM.

### BCL-X_L_ inhibition combined with hydroxychloroquine potently suppresses MPM growth in vivo

**Fig. 6:**
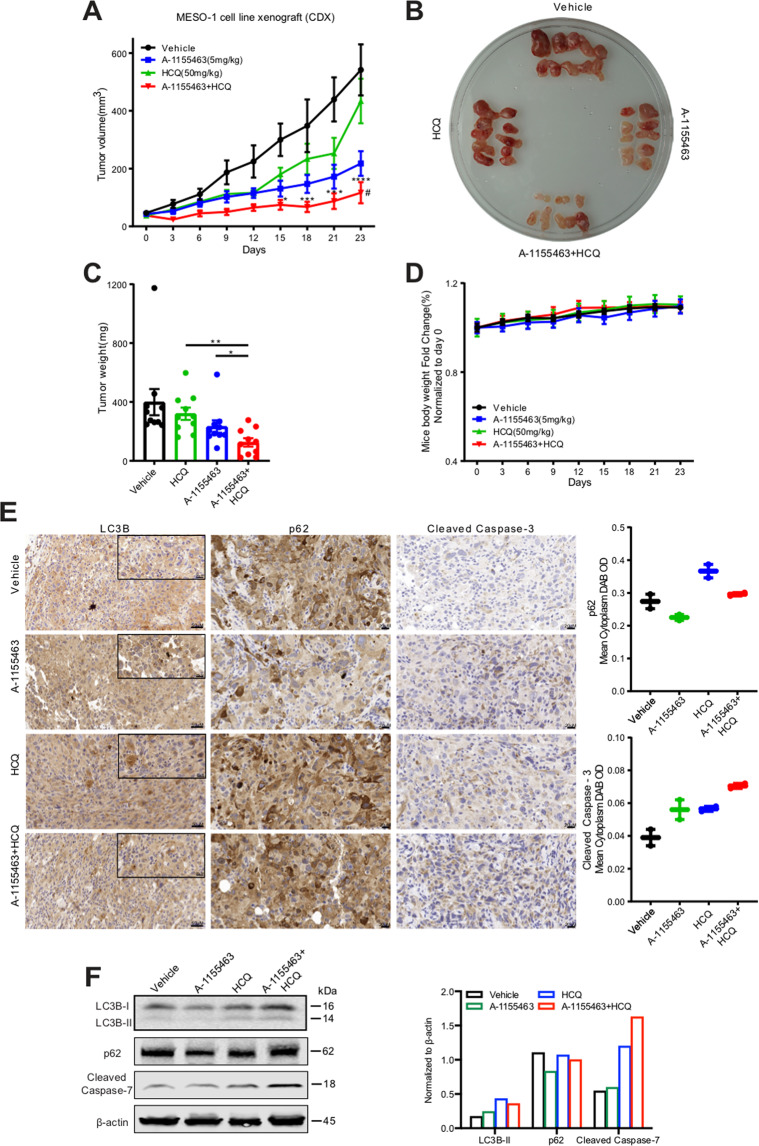
Hydroxychloroquine (HCQ) potentiates anti-MPM efficacy of A-1155463 in vivo. **A** Development of MESO-1 xenograft tumors treated with the indicated drugs. Data were presented as mean ± SEM (*n* = 5). ^#^*p* < 0.05, comparison between combination (A-1155463 plus HCQ)- versus A-1155463-treated group by two-way ANOVA with Tukey’s multiple comparisons test. *p < 0.05, **p < 0.01, and **** *p* < 0.001, comparison between combination- versus HCQ-treated group by two-way ANOVA with Tukey’s multiple comparisons test. **B**, **C** Tumor size (**B**) and weights (**C**) after the treatment. Data were presented as mean ± SEM (*n* = 5). *p < 0.05 and ** *p* < 0.01 by unpaired *t*-test. **D** Mice body weights during the treatment. Data were presented as mean ± SEM (*n* = 5). **E** Immunohistochemical staining for LC3B, p62, and cleaved caspase-3 of MESO-1 xenograft tumors after the treatment. Quantification of p62 and cleaved Caspase-3 in the entire tissue sections were performed by QuPath. **F** Immunoblots for LC3B, p62, and cleaved caspase-7 of MESO-1 xenograft tumors after the treatment. Protein quantification is shown to the right.

To extend the in vitro observations to in vivo, we evaluated efficacy of the combination treatment with A-1155463 and HCQ in MESO-1 xenografts. Whereas A-1155463 alone delayed tumor growth, the addition of HCQ profoundly enhanced antitumor efficacy without obvious side effects, i.e., body weight loss (Fig. 6A–D). Immunohistochemical analysis demonstrated that tumors treated with A-1155463 alone showed enhanced punctate staining of LC3B (highlighted in insets) and reduced p62 compared with the vehicle group (Fig. 6E), consistent with an increase in the autophagic flux upon BCL-X_L_ inhibition as we showed in vitro. Importantly, the combination treatment blunted A-1155463-elicited autophagy, accompanied by increase in tumor cell apoptosis as indicated by the increase in cleaved caspase-3 in the combination group compared with single treatment (Fig. 6E). Immunoblots indicated that drug combination of A-1155463 and HCQ increased p62 and cleaved caspase-7 compared with single agents alone (Fig. 6F), further supporting the notion that combined A-1155463 and HCQ suppresses autophagy and induces apoptosis. Overall, these in vivo data validate a novel therapeutic strategy by combined inhibition of BCL-X_L_ and autophagy to target MPM.

## Discussion

In this study, we showed that MPM capitalizes on BCL-X_L_ for anti-apoptosis, and that *NF2/LATS1/2*-alterations and pro-apoptotic gene expression are associated with sensitivity to BCL-X_L_ inhibition. We further revealed that BCL-X_L_ blockage elicited protective autophagy, such that combined treatment with BCL-X_L_-selective BH3 mimetic and clinically approved autophagy inhibitor yields strong and synergistic anti-MPM effects in vitro and in vivo. Our data suggest the therapeutic potential of targeting BCL-X_L_ alone for MPM subsets, and of co-targeting autophagy for unselected MPM.

Apoptosis is regulated by pro- and anti-apoptotic BCL-2 proteins, which is invariably deregulated in cancer^11,13^. In response to oncogenic or stress signals, malignant cells overexpress anti-apoptotic proteins to dampen apoptosis by sequestering pro-apoptotic activators^35^. In this scenario, cancer cells are proposed to be “primed” for apoptosis, as they accumulate sufficient amounts of the pro-apoptotic activators^36^, which has engendered the concept of cancer treatment by conquering or overwhelming anti-apoptotic defenses, e.g., blockage of specific or multiple pro-survival proteins with BH3 mimetics such as ABT-263 (navitoclax) and ABT-199 (venetoclax)^12,42^. In MPM, apoptosis suppression was reported to be promoted by defects in core-apoptosis signaling^43^, and the pro-apoptotic BH3 mimetic ABT-737 targeting BCL-2/BCL-X_L_/BCL-W^44^ and a pan-BCL-2 inhibitor (JY-1-106) were active against MPM cells^45,46^. However, despite the promising clinical activity of pan-BH3 mimetic drugs, challenges still prevail due to intrinsic or/and acquired resistance and on-target platelet toxicity^14,47–49^, necessitating the need to dissect the survival dependency on individual BCL-2 proteins and the use of selective BH3 mimetics in clinical development. We show here that BCL-X_L_ is a major survival dependency for MPM cells and that the BCL-X_L_ by selective BH3 mimetic demonstrates therapeutic potential for subsets of MPM. Notably, our data are consistent with earlier observations that antagonizing BCL-X_L_ by alternative strategies (e.g., antisense oligonucleotides) suppresses MPM cell survival^44,50^ and with the finding of a very recent study^51^ published amid the manuscript preparation of our work.

We showed for the first time that BCL-X_L_ inhibition elicits protective autophagy that limits the efficacy of BCL-X_L_-selective BH3 mimetics. Autophagy and apoptosis constitute two important self-destructive processes to maintain cellular homeostasis^52^, and there is a complex reciprocal interplay^53–55^. Apoptosis activation can either increase or decrease autophagy, but the underlying mechanisms are controversial^56^. Recent studies have reported that inhibition of pro-survival BCL-2 proteins with BH3 mimetics could induce autophagy either by releasing Beclin-1 from the BH3-binding groove of BCL-2/BCL-X_L_ or by BAX- and BAK1-mediated LC3B lipidation^57–59^. In line with this notion, we revealed that targeting autophagy with HCQ synergistically enhances the cytotoxic effect of A-1155463, suggesting that this combination may be a novel strategy for treating MPM. Notably and in further support of our findings, a recent report published amid the revision of this study showed that BCL-X_L_ is overexpressed and is an important pro-survival protein in MPM cells^60^.

The lack of therapeutically exploitable mutations has significantly hampered the development of targeted therapies for MPM^2,6^, which, however, highlights the importance to identify oncogenic dependencies rather than specific driver mutations to combat MPM^1^. We have systematically assessed the pro-survival BCL-2 proteins for their contributions to anti-apoptosis in MPM cells, which, to the best of our knowledge, has remained incompletely defined. Overall, our work demonstrates the therapeutic potential of BCL-X_L_-specific BH3 mimetics in MPM, alone and in combination with the FDA-approved HCQ.

## Supplementary information

Supplementary Information

Supplementary Figure 1

Supplementary Figure 2

Supplementary Figure 3
