# Supplementary material for: Malignant pleural mesothelioma co-opts BCL-XL and autophagy to escape apoptosis
Source: Cell Death Dis. 2021 Apr 15;12(4):406. doi: 10.1038/s41419-021-03668-x (PMC8050302; doi:10.1038/s41419-021-03668-x)
Supplement: Supplementary file 1 — Supplementary Information [file 41419_2021_3668_MOESM1_ESM.docx]

**SUPPLEMENTARY FIGURE LEGENDS**

**Figure S1. BCL-X_L_ expression is increased in MPM cells.**

**A**, Immunoblots of BCL-X_L_ in human normal fibroblasts (hFb16Lu), normal mesothelial cells (LP-9) and MPM cells.

**B,** Quantification of BCL-X_L_ normalized against Actin. Data were from two independent experiments (n=2).

**Figure S2. BCL-X_L_ as a therapeutic target in MPM.**

**A**, Cell viability assay of LP-9 and MPM cells treated with the BCL-2 inhibitor venetoclax for 96 h. Data are presented as mean ± s.d. (n=2).

**B**, Immunoblots of BCL-X_L_ and BAX in the indicated MPM cell lines.

**C**, Immunoblots of LP-9 cells pre-treated with A-1155463 (1 μM) for 24h and subsequently exposed to Bafilomycin A1 (200 nM) for additional 2h. Quantification of LC3B-II normalized against β-actin is shown.

**Figure S3. Concurrent blockage of BCL-X_L_ and autophagy yields synergistic anti-MPM effects.**

**A**, Kaplan–Meier analysis of MPM based on BCL-X_L_ protein level. TCGA cohort of MPM patients (n=61) with high- (in red) or low- BCL-X_L_ (in black) were stratified by optimal cutoff value of the BCL-X_L_ across all patients using the surv_cutpoint function in the R “maxstat” package. The p-value is calculated by the log-rank test using R (version 3.4.3).

**B**, Dose-response curves of LP-9 cells treated with A-1155463 and HCQ, alone or in combinations for 96 h. Data are shown as mean ± s.d. (n=3).

**C, D,** Dose-response curves (C) of MPM cells treated with ABT-263 and HCQ, alone or in combination for 96 h. Data are shown as mean ± s.d. (n=3). Combination index (D) values were determined by the CompuSyn software. CI<1.0, synergism; CI=1.0, additive effects, CI>1.0, antagonism.

**E,** Clonogenic assay of MPM cells treated with ABT-263 and HCQ, alone or in combination for 96 h. The cells were further cultured for 10 days before crystal violet staining. Representative images of two independent experiments (n=2) are shown.
