# Supplementary figures and images for: Malignant pleural mesothelioma co-opts BCL-XL and autophagy to escape apoptosis
Source: Cell Death Dis. 2021 Apr 15;12(4):406. doi: 10.1038/s41419-021-03668-x (PMC8050302; doi:10.1038/s41419-021-03668-x)

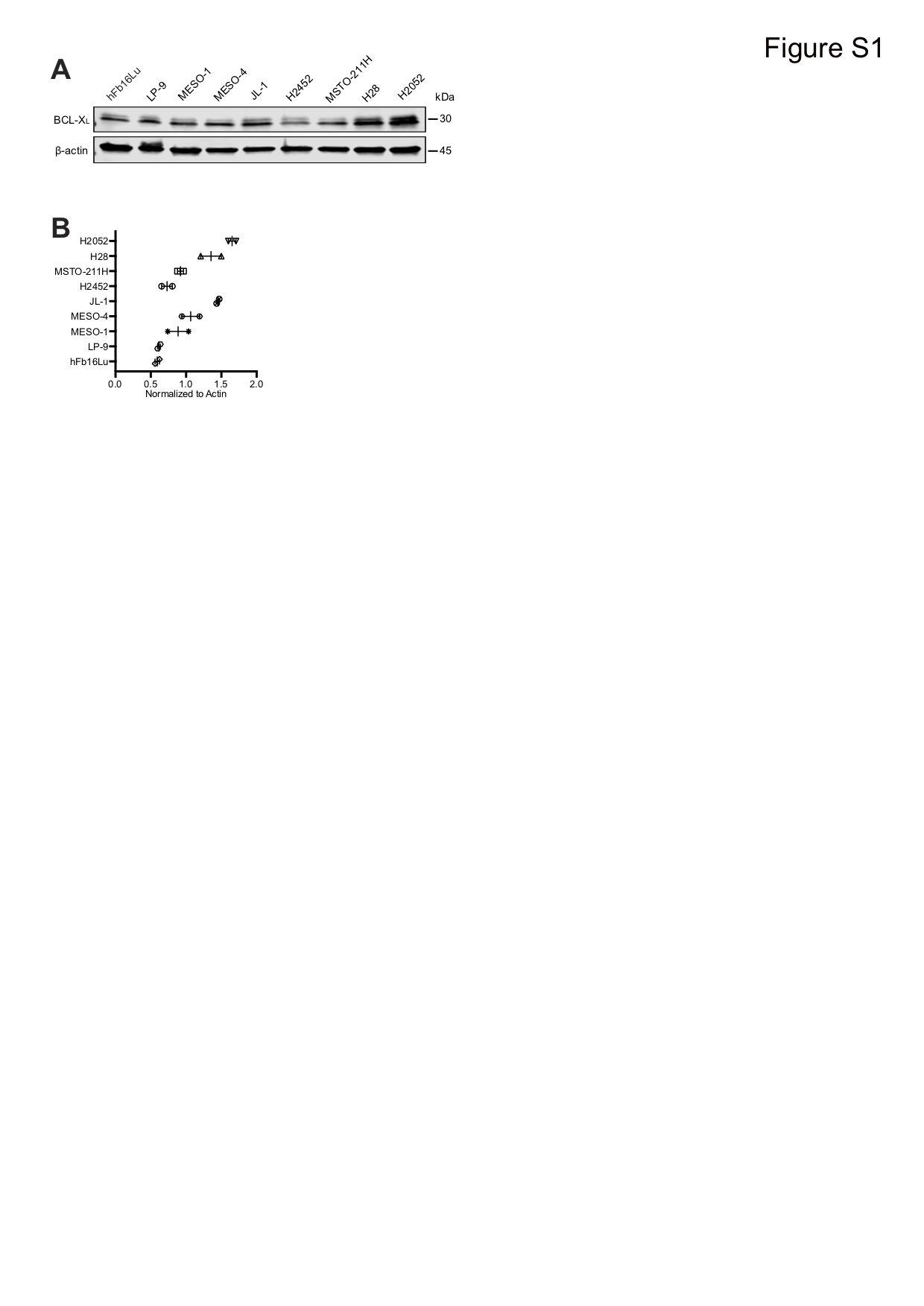

Supplement: Supplementary file 2 — Supplementary Figure 1 [file 41419_2021_3668_MOESM2_ESM.png]

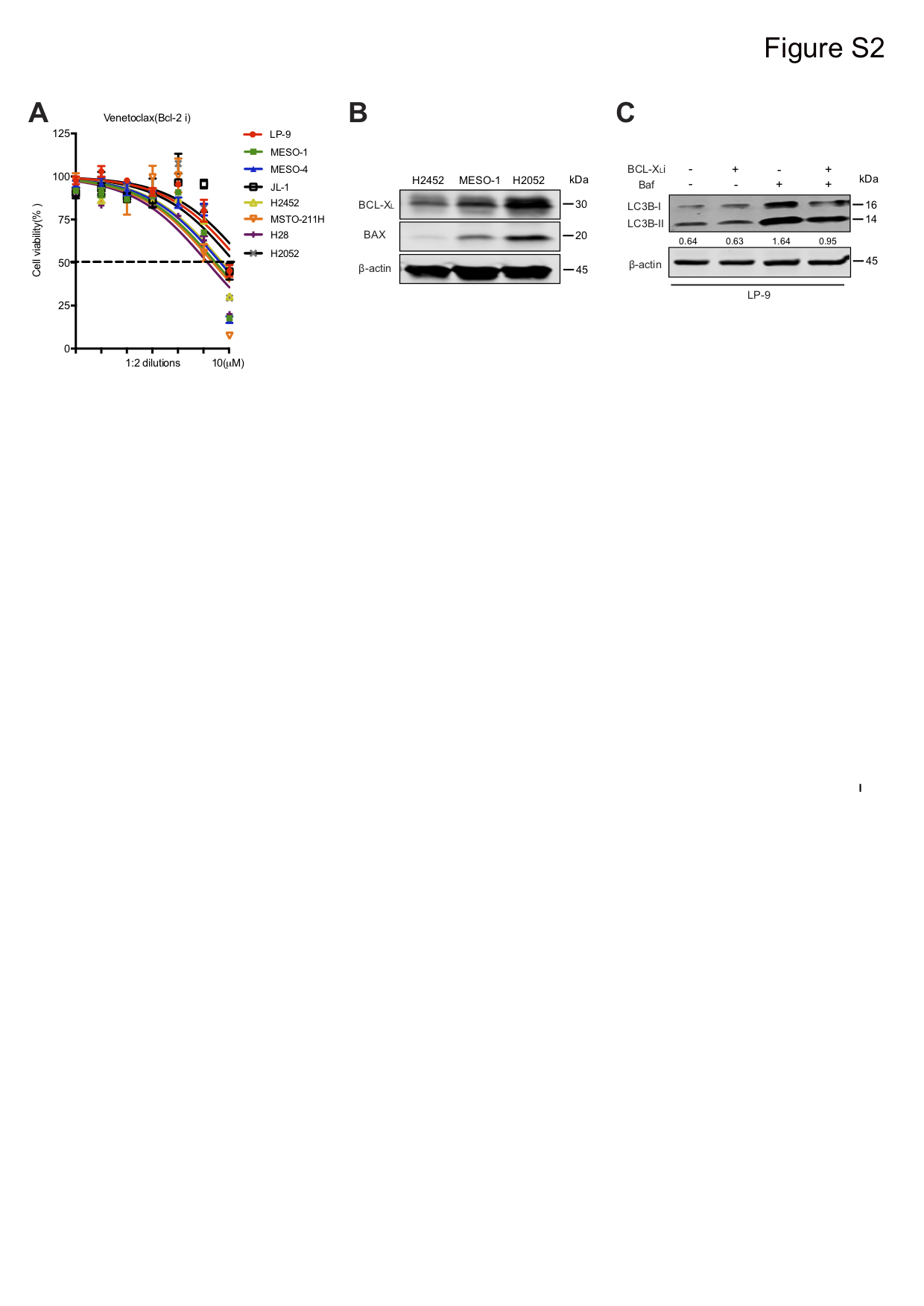

Supplement: Supplementary file 3 — Supplementary Figure 2 [file 41419_2021_3668_MOESM3_ESM.png]

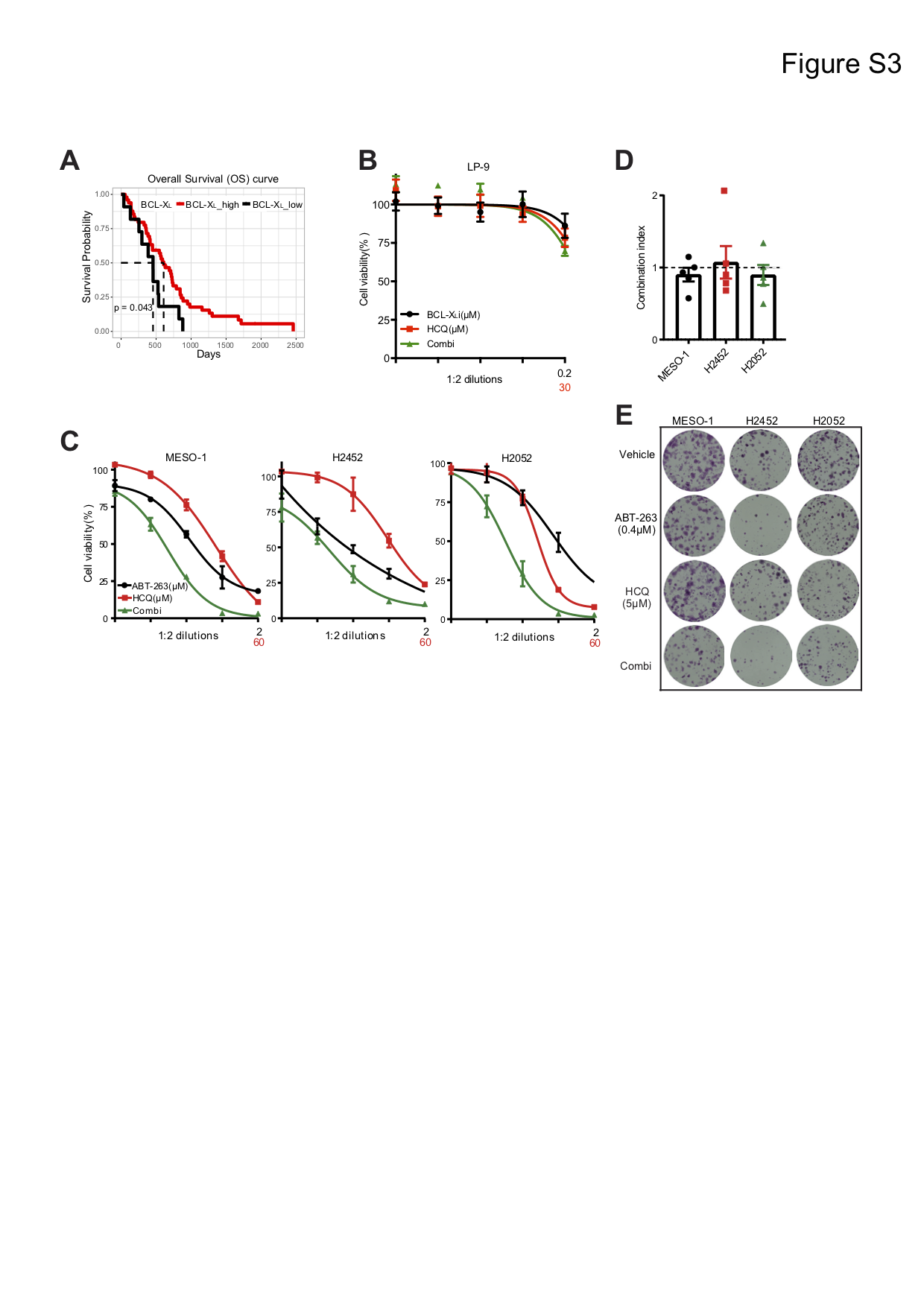

Supplement: Supplementary file 4 — Supplementary Figure 3 [file 41419_2021_3668_MOESM4_ESM.png]
